# Transfusion von Plasmaprodukten: ein Update für Anästhesisten

**DOI:** 10.1007/s00101-025-01564-z

**Published:** 2025-07-22

**Authors:** Dominik Jenny, Romana Erblich, Bernhard Eichler, Susanne Süßner, Alexander Weigl, Jens Meier, Martin W. Dünser

**Affiliations:** 1 Klinik für Anästhesiologie und Intensivmedizin, Kepler Universitätsklinikum und Johannes Kepler Universität, Krankenhausstraße 9, 4020 Linz, Österreich; 2 Blutzentrale Linz, Oberösterreichisches Rotes Kreuz, Linz, Österreich; 3 Anstaltsapotheke, Kepler Universitätsklinikum und Johannes Kepler Universität, Linz, Österreich

**Keywords:** Gerinnung, Volumenersatz, Hämorrhagischer Schock, Blutung, Trauma, Sepsis, Plasma, Transfusion, Coagulation, Volume substitution, Hemorrhagic shock

## Abstract

In der Geschichte wurde Plasma zunächst als Volumenersatzmittel eingesetzt, später dann zur Gerinnungssubstitution, jedoch mit eingeschränkter Wirksamkeit. Als Volumenersatzmittel scheinen Plasmaprodukte im hämorrhagischen Schock geeignet und kristalloiden Infusionslösungen überlegen zu sein, um das Outcome von massiv blutenden (Trauma‑)Patienten zu verbessern. Verschiedene Plasmaprodukte befinden sich derzeit auf dem Markt und bieten unterschiedliche Vor- und Nachteile, weshalb die Auswahl sorgfältig getroffen werden muss. Die Transfusion von Plasmaprodukten soll AB0-gleich, in Notfällen kann sie auch AB0-kompatibel erfolgen. An häufige Nebenwirkungen wie die iatrogene Hypokalzämie und die transfusionsassoziierte Hypervolämie muss gedacht werden. Die Hauptindikation zur Transfusion von Plasmaprodukten für Anästhesisten liegt in der Behandlung von hämorrhagischen Schockzuständen sowie in Ausnahmefällen in der Substitution seltener Einzelfaktorenmängel. In der Praxis gibt es jedoch häufig Fehlindikationen, weswegen die Anwendung vor jeder Plasmatransfusion kritisch überprüft werden soll.

## Abstract (en)

Historically, plasma was first used as volume replacement and later for coagulation substitution, although with limited effectiveness. As almost coagulation neutral volume replacement agents, plasma products are useful in treating patients suffering from traumatic hemorrhagic shock and seem to be superior to crystalloid infusion solutions for improving the outcome. Various plasma products are currently available on the market, each offering different advantages and disadvantages, which necessitates careful selection. The transfusion of plasma products should have the same AB0 group but in time critical emergencies AB0-compatible plasma can also be used. The previously feared risk of infection has been significantly reduced thanks to improved donor selection, testing and manufacturing processes, so that today other side effects are more prominent. Common side effects, such as iatrogenic hypocalcemia and transfusion-associated circulatory overload, must be considered. Careful evaluation of the correct indications for plasma transfusion can greatly reduce the occurrence of adverse events. For anesthesiologists the main indications for transfusion of plasma products are the treatment of hemorrhagic shock and, in exceptional cases, the substitution of rare individual factor deficiencies; however, in practice, there are often incorrect indications, which is why the administration should be critically reviewed before each plasma transfusion. The current literature no longer recommends the prophylactic use of plasma prior to procedures (e.g., tracheostomy, central venous catheter placement) in patients with abnormal coagulation tests but no evidence of bleeding. Patients with (chronic) liver dysfunction and abnormal coagulation parameters but without clinical correlates, should also not receive transfusions. Further research is still required to determine whether plasma plays a role in sepsis and in patients suffering from severe burns.

## Einleitung

Etwa 55 % des im Körper zirkulierenden Blutvolumens bestehen aus Plasma. Dieses dient primär als Transportmedium für zelluläre Bestandteile, Proteine sowie niedermolekulare Substanzen wie Elektrolyte und Glucose. Plasma steht in ständigem Austausch mit der endothelialen Glykokalyx. Diese wird durch die Zusammensetzung des Plasmas und der infundierten Flüssigkeiten direkt beeinflusst. Die Glykokalyx reguliert die Gefäßpermeabilität, beeinflusst die Blutgerinnung, moduliert Entzündungsreaktionen und schützt das Endothel vor mechanischen Stressoren. Derzeit gibt es verschiedene Plasmapräparate für den medizinischen Einsatz. Trotz des Rückgangs von Plasmatransfusionen spielt Plasma weiterhin eine wichtige Rolle in der modernen Medizin, wobei sich die Indikationen im Laufe der Zeit verändert haben. In diesem Artikel soll auf die klinische Historie des Plasmas, die unterschiedlichen Arten sowie Nebenwirkungen und die wichtigsten Indikationen für Anästhesisten eingegangen werden.

## Geschichte

Ab 1936 gewann Plasma als Volumenersatz bei traumatischen Verletzungen zunehmend an Bedeutung. Während zuvor Vollblutkonserven zur Kompensation des Blutverlusts genutzt wurden, erwies sich Plasma als vorteilhafter, da es länger haltbar und einfacher zu lagern war.

Im Laufe der Geschichte, insbesondere während kriegerischer Auseinandersetzungen, traten jedoch auch die Schattenseiten von Plasma zutage: Verunreinigungen während der Herstellung und die Übertragung von Hepatitis stellten erhebliche Risiken dar. In Folge galten kristalloide und kolloidale Lösungen als vielversprechende Alternativen, führten jedoch zur Entstehung neuer Krankheitsbilder, wie der „Da-Nang lung“, die später als Acute Respiratory Distress Syndrome (ARDS) bekannt wurde.

Anfang der 1990er-Jahre wurde mit der Einführung des Solvent-Detergent-Verfahrens bei der Plasmaherstellung ein bedeutender Fortschritt erzielt. Durch Optimierung des Herstellungsprozesses sowie eine gezielte Spenderauswahl konnte die Sicherheit von Plasma erheblich verbessert werden, sodass Plasma wieder in den klinischen Alltag integriert wurde – diesmal jedoch nicht als Volumenersatz, sondern als Gerinnungsprodukt. Aufgrund neuer wissenschaftlicher Erkenntnisse sowie der zunehmenden Verfügbarkeit von einzelnen Gerinnungsfaktoren und Kombinationspräparaten verschob sich die Indikation für Plasma jedoch deutlich und wurde zunehmend auf selektive Einsatzbereiche beschränkt.

## Herstellung von Plasmaprodukten

Plasma kann aus Vollblut oder mittels Plasmapherese hergestellt werden. Bei beiden Methoden wird Plasma durch Zentrifugation von den anderen Blutbestandteilen getrennt. Bei der Plasmapherese werden die zellulären Blutkomponenten wieder refundiert. Somit können größere Mengen an Plasma gewonnen werden – je nach Gewicht des Spenders bis zu 880 ml [1].

Bei Plasmaprodukten wird nur die Aktivität des Gerinnungsfaktor VIII überprüft. Im Fall einer Einzelspende muss eine Aktivität ≥ 70 % nach dem Auftauen vorhanden sein; bei gepooltem Plasma muss diese mindestens 0,7 U/ml betragen [1].

Sowohl Faktor VIII als auch Fibrinogen sind Akute-Phase-Proteine. Deshalb kann die Konzentration bei Spendern erheblich schwanken. Besonders hoch ist diese Schwankungsbreite daher bei Einzelspenderplasma.

### Arten von Plasmaprodukten

Plasma kann auf verschiedene Weisen verarbeitet werden, wobei das Verfahren und die Lagerungsbedingungen die verbleibende Aktivität der Gerinnungsfaktoren beeinflussen (Tab. 1). Generell gilt: Je niedriger die Lagerungstemperatur, desto länger die Haltbarkeit des Plasmas. Eine Ausnahme ist lyophilisiertes Plasma, welches bei Raumtemperatur bis zu 24 Monate haltbar ist. Niedrigere Lagerungstemperaturen erhöhen allerdings den logistischen Aufwand und verlängern die Vorbereitungszeit vor der Transfusion, was in Notfällen die zeitnahe Transfusion kritisch verzögern kann.

**Tab. 1:** Überblick über verschiedene Plasmaprodukte. (Quelle: [1, 44])

Plasma-Arten	Herstellung	Lagerung	Haltbarkeit	Vorbereitung	Gerinnung
*FFP*	Innerhalb 6 h nach Abnahme Kühlung auf < −25 °C	< −25 °C	36 Monate	45 min	> 70 % Faktor VIII des Ausgangswertes
*FP 24*	Innerhalb 24 h nach Abnahme Kühlung auf < −25 °C	< −25 °C	12 Monate	45 min	65–80 % Faktor VIII des Ausgangswertes; reduziertes Protein C
*Aufgetautes Plasma*	Nach Auftauen	2–6 °C	5 Tage	0 min	Reduzierter Faktor V und Faktor VIII
*Flüssiges Plasma*	Noch nie gefroren	2–6 °C	5 Tage	0 min	Physiologische Konzentration aller Gerinnungsfaktoren
*Lyophilisiertes Plasma*	Entzug der Flüssigkeit im Vakuum	+2 bis +25 °C	15–24 Monate	Ca. 6 min	> 70 % Faktor VIII des Ausgangswertes; 75–80 % Faktor VII und vWF

*FFP* Fresh Frozen Plasma, *FP24* Frozen Plasma innerhalb von 24 h

### Virusinaktivierung

Im Vergleich zu Erythrozyten- oder Thrombozytenkonzentraten besteht bei Plasmaprodukten ein potenziell höheres Infektionsrisiko. Dieses ist bedingt durch die diagnostische Lücke und der deutlich höheren Spendenfrequenz (Anmerkung: Plasma kann bis zu 60-mal (Deutschland) [2] bzw. 50-mal (Österreich) [3] pro Jahr gespendet werden, während Erythrozyten nur 6‑mal pro Jahr gespendet werden dürfen). Durch stetig sensitivere PCR-Testungen kann diese diagnostische Lücke zwar verkleinert, jedoch nicht gänzlich eliminiert werden. Zusätzlich spielen neue Krankheitserreger, „emerging pathogens“, eine wichtige Rolle in der Transfusionsmedizin. Dabei handelt es sich um neu entdeckte Infektionserreger (SARS-CoV-2) oder aufgrund von veränderten Umwelt- oder sozialen Faktoren (Reiseverhalten) vermehrt auftretende Erreger (beispielsweise West-Nil‑, Dengue-Virus, Plasmodien). Aus diesen Gründen mussten zusätzliche Methoden entwickelt werden:

- *Quarantänelagerung:*
  Ziel ist die Schließung der diagnostischen Lücke. Der Spender wird auf humanes Immundefizienz‑, Hepatitis-B-, Hepatitis-C-Virus etc. getestet. Wenn diese Tests negativ sind, wird das Plasma für 4 Monate bei −30 °C in Quarantäne gelagert. Nach dieser Frist muss der Spender erneut getestet werden. Erst, wenn alle Tests negativ sind, wird das Plasma zur Transfusion freigegeben.
- *Solvent/Detergent(S/D)-Behandlung:*
  Bei diesem Verfahren werden Viren inaktiviert. Dazu werden ca. 500 bis 1600 Plasmen gepoolt. Durch Zugabe eines Solvens und Detergens wird die Lipidhülle der Viren aufgebrochen und das Virus zerstört. Spender müssen mit PCR-Tests auf Viren ohne Lipidhülle getestet werden.
- *Pathogenreduktion mittels Zusatzstoffen und Bestrahlung:*
  Durch Zugabe von Methylenblau und monochromatischem Licht wird die DNA bzw. RNA von Viren, Bakterien und Parasiten aufgebrochen und inaktiviert.

Alle Formen der Virusinaktivierung verringern in unterschiedlichem Ausmaß die Aktivität der Gerinnungsfaktoren. Diese Reduktion ist bei pathogenreduziertem Plasma am höchsten, da es bei diesem Verfahren zu einer ca. 35 %igen Reduktion der Konzentration/Aktivität von Fibrinogen, Faktor V und Faktor VIII kommt [5].

## Transfusionspraxis

Plasma muss über ein Infusionsbesteck mit Standardfilter (Porengröße 170–230 μm) verabreicht werden. Dieses sollte mindestens alle 6 h gewechselt werden [4]. Gerade bei FFP ist auf eine rasche Verabreichung zu achten, da die Gerinnungsfaktoren nach dem Auftauen mit der Zeit deutlich an Wirksamkeit verlieren: Der Faktor VIII ist für 2 h stabil, der Faktor VII für 5 h und die restlichen Gerinnungsfaktoren für zumindest 6 h [5].

Grundsätzlich soll Plasma AB0-gleich transfundiert werden. In Notfallsituationen kann jedoch auf ein AB0-kompatibeles Plasma ausgewichen werden; dies entspricht heutzutage der gängigen Praxis [2]. Im Gegensatz zu Erythrozytenkonzentraten gilt bei Plasma die Blutgruppe AB als Universalspender und die Blutgruppe 0 als Universalempfänger (Tab. 2). Da die Blutgruppe AB bei Menschen in Mitteleuropa jedoch nur zu etwa 4 % vorkommt, sollte dieses Universalplasma mit Bedacht eingesetzt werden [4]. Es konnte gezeigt werden, dass bei fehlendem AB-Plasma auch Plasma der Blutgruppe A mit niedrigem Anti-B-Titer eine geeignete Alternative darstellen kann [6].

**Tab. 2:** Gegenüberstellung von Patientenblutgruppe, Plasmagleichheit und Plasmakompatibilität

Patientenblutgruppe	AB0-Gleichheit	AB0-Kompatibilität
*A*	A	A und AB
*B*	B	B und AB
*AB*	AB	AB
*0*	0	0, A, B und AB

Bei AB0-kompatibler Plasmatransfusion zeigt sich kein Mortalitätsunterschied, jedoch eine erhöhte Rate an Nebenwirkungen (53,5 % vs. 40,5 %, *p* = 0,002). Je höher die Menge des verabreichten Volumens ist, desto mehr steigt auch die Komplikationsrate. Bei Verabreichung von > 6 Einheiten liegt das Risiko bei 70 %. Das größte Risiko für Nebenwirkungen haben Empfänger der Blutgruppe 0 [7].

## Nebenwirkungen

Die Risiken der Nebenwirkungen von Plasmatransfusionen sind über die letzten Jahre immer geringer geworden. Das gefürchtete Infektionsrisiko konnte dank besserer Spenderauswahl, Test- und Herstellungsverfahren deutlich reduziert werden, sodass heute andere Nebenwirkungen im Vordergrund stehen (Tab. 3).

**Tab. 3:** Überblick über Nebenwirkungen bei Plasmatransfusionen. (Quelle: [10, 11, 13])

	Definition	Pathophysiologie	Auftreten	Prävention	Therapie
*Iatrogene Hypokalzämie*	Ionisiertes Kalzium < 1,15 mmol/l	Zitrat in Plasmaprodukten bindet Kalzium im Blut	Keine Daten	Kontrolle ionisierter Kalziumspiegel	Intravenöse Kalziumsubstitution
*TRALI*	Akute Lungeninsuffizienz innerhalb 6 h nach Transfusion; nichtkardiales Lungenödem ohne andere Ursache	HLA-Antikörper aktivieren neutrophile Granulozyten in Lunge → gesteigerte Permeabilität → Lungenödem	1:5000–1:10.000	Geeignetes Plasmaprodukt: S/D-Plasma, HLA-Antikörper-Screening vor Plasmaspende	Symptomatisch: Sauerstoffgabe, Intubation, ECMO
*TACO*	Zeichen generalisierter Hypervolämie innerhalb 12 h nach Transfusion	Erhöhtes intravasales Volumen → Anstieg venöser Drücke mit Rückstau	1,5–11 % aller Transfusionen	Geeignete Indikationsstellung und Vorsicht bei Patienten mit Herz‑, Leber- oder Niereninsuffizienz	Diuretikagabe, zusätzlich symptomatische Therapie
*Allergische Reaktionen*	Potenziell lebensbedrohliche systemische Immunreaktion	Allergische Reaktion auf Plasma, Plasmabestandteile oder enthaltene Konservierungsstoffe	Schwere Reaktionen: 1:18.000–1:172.000	Keine Gabe bei bekannter Allergie	Symptomatische Behandlung je nach Schweregrad
*TRIM*	Immunologische Veränderungen durch Transfusion	Leukozyten sowie deren Zerfallsprodukte stimulieren Immunsystem	Keine Daten	Verwendung von leukozytendepletierten, pathogenreduzierten Produkten	Keine spezifische Therapiemöglichkeit

Die beste Möglichkeit zur Vermeidung von Nebenwirkungen ist die kritische Indikationsstellung und sparsame Anwendung von Plasma!

*TRALI* „transfusion-related acute lung injury“, *HLA* humanes Leukozytenantigen, *S/D* Solvent/Detergent, *ECMO* extrakorporale Membranoxygenierung, *TACO* „transfusion-associated circulatory overload“, *TRIM* „transfusion-related immunomodulation“

Im Jahr 2022 wurden insgesamt 1.893.000 Plasmatransfusionen im europäischen Raum durchgeführt. Dabei traten 176 wahrscheinliche oder gesicherte schwere unerwünschte Nebenwirkungen auf. Mit einer Inzidenz von 9,1/100.000 Transfusionen liegt das Risiko somit zwischen dem von Erythrozytenkonzentraten (6,4/100.000) und von Thrombozytenkonzentraten (19,8/100.000). Bei 67 Patienten trat eine Anaphylaxie bzw. eine Hypersensitivitätsreaktion auf. In 10 Fällen kam es zu einer transfusionsassoziierten kardialen Überladung, und in 6 Fällen wurde eine Hämolyse aufgrund einer AB0-Inkompatibilität beobachtet. In 2 Fällen wurde ein letaler Ausgang nach Plasmatransfusionen beobachtet [8].

### Iatrogene Hypokalzämie

Von allen Blutprodukten enthalten FFP am meisten Zitrat, weswegen die ionisierte Kalziumkonzentration gerade bei der Transfusion von Plasmaprodukten regelmäßig kontrolliert und bei Bedarf durch i.v.-Kalzium-Gaben (z. B. mittels Kalziumglukonat 10 %) angehoben werden muss. In einer Sekundäranalyse zweier präklinischer Studien bei Patienten im hämorrhagischen Schock konnten Moore et al. nachweisen, dass eine Hypokalzämie bei Traumapatienten nach Plasmagabe häufiger auftrat (53 % bei Plasmagabe und 36 % in der Kontrollgruppe, *p* = 0,03). Diese war ebenso mit einer signifikant niedrigeren Überlebensrate verbunden, während die Rate an Massivtransfusionen anstieg [9].

### „Transfusion-related acute lung injury“

Die „Transfusion-related acute lung injury“ (TRALI) wird als akute Atemnot innerhalb von 6 h nach der Transfusion eines Blutprodukts definiert. Pathophysiologisch liegt ein nichtkardiogenes Lungenödem ohne andere erklärbare Ursache vor [10]. Jahrelang war TRALI die häufigste Ursache aller transfusionsbedingten Todesfälle. Mittlerweile ist sie nur noch für 4 % der Fälle verantwortlich. Eine TRALI kann bei der Transfusion sämtlicher Blutprodukte auftreten; relativ gesehen, tritt sie jedoch am häufigsten bei Plasmatransfusionen auf. Verantwortlich für das Auftreten sind in erster Linie Antikörper gegen humanes Leukozytenantigen (HLA) oder granulozytäre Antikörper im Plasma des Spenders. Die HLA-Antikörper aktivieren beim Empfänger neutrophile Granulozyten im Gefäßbett der Lunge, wodurch Zytokine und reaktive Sauerstoffspezies freigesetzt werden. Diese bewirken eine erhöhte Permeabilität des Lungenendothels, was in der Folge ein Lungenödem verursacht. Therapeutisch ist in milden Fällen eine Sauerstoffgabe ausreichend. In schweren Fällen kann hingegen die Intubation mit invasiver Beatmung oder sogar eine extrakorporale Membranoxygenierung erforderlich sein. Um das TRALI-Risiko zu minimieren, gibt es verschiedene Ansätze: Plasmaspenden nur von Männern oder von Frauen, die keine Schwangerschaft durchgemacht haben bzw. ein negatives HLA-Antikörper-Screening aufweisen. Das Herstellungsverfahren beeinflusst das TRALI-Risiko: Da S/D-Plasma aus dem Plasma vieler Spender gemischt (=gepoolt) wird, werden eventuell vorhandene HLA-Antikörper stark verdünnt. Dadurch ist das Risiko für TRALI bei S/D-Plasma besonders niedrig [11].

### „Transfusion-associated circulatory overload“

Der „Transfusion-associated circulatory overload“ (TACO) präsentiert sich klinisch ähnlich wie das TRALI, wodurch die Unterscheidung der beiden Syndrome erschwert wird. Pathophysiologisch liegt dem TACO eine Hypervolämie mit Lungenödem infolge erhöhter pulmonalvenöser Drücke infolge der Plasmatransfusion zugrunde. Daher ist bei diesen Patienten eine Diuretikagabe angezeigt. Die Inzidenz des TACO liegt bei 1,5–11 %. Besonders anfällig sind kritisch kranke und ältere Patienten, insbesondere wenn sie an einer Herzinsuffizienz, schweren Nieren- oder Leberschädigung leiden. In einer Hämovigilanzstudie aus England war der TACO die häufigste Ursache von transfusionsbedingten Todesfällen (44,1 % der Fälle) [11].

Die Reversierung von Vitamin-K-Antagonisten oder direkten oralen Antikoagulanzien (DOAK) mittels Plasma erfordert meist große Volumina (30 ml/kgKG). Da bei diesen Patienten meist kein Volumenmangel besteht und sie häufig eine eingeschränkte kardiovaskuläre Reserve aufweisen, erhöht die zusätzliche Flüssigkeitsbelastung das Risiko für einen TACO erheblich. Aus diesem Grund ist die Plasmagabe bei diesem Krankheitsbild obsolet. Stattdessen soll die Antagonisierung von Vitamin-K-Antagonisten mit Prothrombinkomplexkonzentraten erfolgen. Bei Patienten unter Einnahme von DOAK können spezifische Antidote (z. B. Idarucizumab bei Dabigatran oder Andexanet alfa bei Faktor-Xa-Hemmern) oder ebenfalls Prothrombinkomplexkonzentrate angewendet werden. Aufgrund der unsicheren Datenlage zu Andexanet alfa sowie möglicher Nachteile (z. B. erhöhtes Thromboembolierisiko, kurze Halbwertszeit, hohe Kosten) sollte die Antagonisierung von Faktor-Xa-Hemmern im Rahmen einer sorgfältigen Einzelfallentscheidung unter kritischer Abwägung von Nutzen und Risiko erfolgen [12].

### Allergische Reaktionen

Allergische Transfusionsreaktionen treten in weniger als 1–3 % aller Transfusionen auf. Die meisten allergischen Reaktionen verlaufen milde und zeigen sich klinisch an Urtikaria, Juckreiz oder Rötung. Manifeste anaphylaktische Reaktionen mit Bronchospasmus, Angioödem und Hypotension sind sehr selten (ca. 1:18.000 bis 1:172.000) [13]. Die Behandlung transfusionsbedingter allergischer Reaktionen erfolgt symptomatisch und orientiert sich am anaphylaktischen Phänotyp, wobei die rechtzeitige Gabe von Adrenalin bei schweren anaphylaktischen Reaktionen von entscheidender Bedeutung ist.

### „Transfusion-related immunomodulation“

Der Terminus Transfusion-related immunomodulation (TRIM) beschreibt die kurz- sowie langfristigen immunologischen Veränderungen, die durch eine Plasma- bzw. Bluttransfusion verursacht werden. Dadurch besteht in der Akutphase ein erhöhtes Risiko für Entzündungsreaktionen, die zur Entwicklung einer Organdysfunktion führen können. In der Sekundärphase kann eine gesteigerte Infektionsanfälligkeit durch immunsupprimierende Effekte begünstigt werden. In einer retrospektiven, multizentrischen Studie aus China wurden eine erhöhte Mortalität (OR 1,05; 95 %-KI; *p* < 0,001) sowie ein gesteigertes Infektionsrisiko nach Plasmagabe festgestellt. Pro 100 ml verabreichtem FFP stiegen im Vergleich zu Patienten ohne FFP-Gabe die Raten für nosokomiale Infektionen (OR 1,03), akutes Atemnotsyndrom (ARDS) (OR 1,03), postoperative Wundinfektionen (OR 1,03) sowie für einen verlängerten Krankenhausaufenthalt (OR 1,05) [14]. Besonders hervorzuheben sind jedoch die äußerst liberale Plasmaverabreichung sowie der Ausschluss von Patienten mit Koagulopathie oder Massivtransfusion. Andererseits gibt es jedoch auch Studien, welche keinen Zusammenhang zwischen einer FFP-Gabe und erhöhtem Infektionsrisiko bei Traumapatienten nachweisen konnten [15].

Obwohl Plasmaprodukte in der Regel leukozytendepletiert sind, können dennoch geringe Mengen an Leukozyten oder deren Zerfallsprodukte das Immunsystem stimulieren. Das Auftreten einer TRIM hängt von der Zusammensetzung des Plasmas, der transfundierten Menge sowie der immunologischen Situation des Empfängers ab. Um die Risiken für das Auftreten einer TRIM zu minimieren, sollten nur leukozytendepletierte und pathogenreduzierte Plasmaprodukte verwendet werden.

## Indikationen zur Plasmatransfusion

Die aktuelle wissenschaftliche Evidenz belegt die Plasmagabe bei Massivblutungen zum Plasmaersatz, zur Substitution der Gerinnungsfaktoren V, XI oder des ADAMTS13 sowie zur Plasmaaustauschbehandlung bei thrombotisch-thrombozytopenischer Purpura [4, 16]. Im Nachfolgenden soll auf die beiden wichtigsten Anwendungsgebiete in der Anästhesie eingegangen werden.

### Plasmaersatz bei Massivblutungen

#### Präklinische Behandlung

**Abb. 1:**
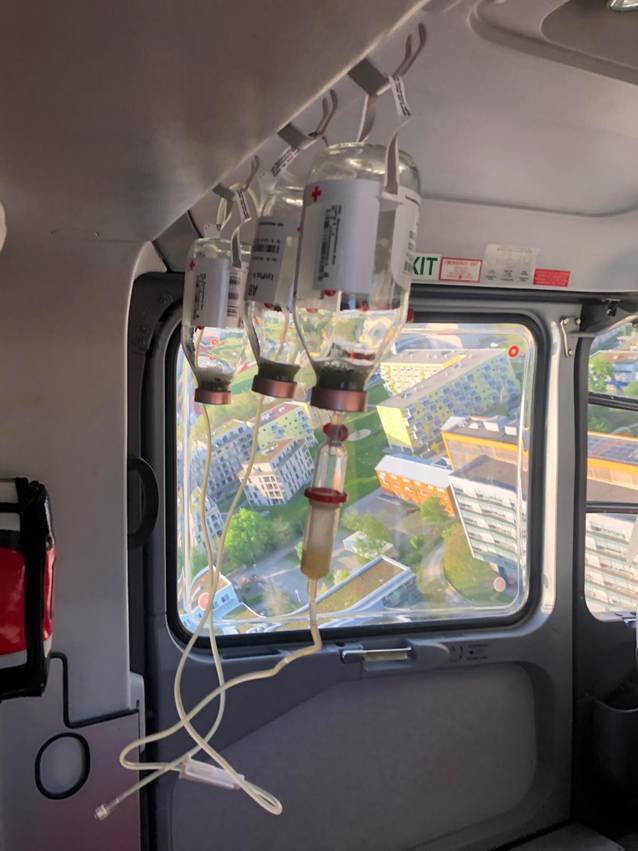
Anwendung von lyophilisiertem Plasma im Notarzthubschrauber zur Behandlung eines Patienten mit hämorrhagischem Schock

Die PAMPer- und COMBAT-Trials untersuchten den Einfluss einer präklinischen Plasmatransfusion auf die Mortalität von Traumapatienten mit hohem Risiko eines schweren Blutverlustes [17, 18]. Trotz ähnlichem Studiendesign kamen beide Studien jedoch zu gegensätzlichen Ergebnissen. Subanalysen beider Trials zeigten, welche Patientengruppen von der prähospitalen Gabe profitieren könnten: solche mit einer präklinischen Versorgungszeit (Zeit am Einsatzort + Transportzeit) über 20 min, Patienten mit stumpfem Traumamechanismus, Polytraumapatienten, Patienten mit begleitendem Schädel-Hirn-Trauma oder bei hämorrhagischem Schock durch kontrollierbare Blutungen. Marginale Unterschiede in den Gerinnungslaborparametern bei Aufnahme der Studienpatienten im Krankenhaus deuten darauf hin, dass der frühe Mortalitätsvorteil durch die Transfusion von Plasma beim Traumapatienten im hämorrhagischen Schock nicht durch gerinnungsaktive Eigenschaften von Plasma bedingt ist, sondern sehr wahrscheinlich durch dessen Volumeneffekt [19]. Lyophilisiertes Plasma könnte im präklinischen Setting aufgrund seiner einfachen Lagerung und der geringen Vorbereitungszeit vorteilhaft sein (Abb. 1). Bei der Interpretation der PAMPer- und COMBAT-Trials ist zu berücksichtigen, dass unabhängig von der jeweiligen Intervention höhere Mortalitätsraten bei vergleichbarem Injury Severity Score (ISS) beobachtet wurden als im deutschen Traumaregister (30-Tages-Mortalität PAMPer: 23,2 % bzw. 33 % bei ISS von 22; Deutsches Traumaregister: < 10 % bei ISS 20–23; RETIC-Trial 7,4 % bei ISS von 34) [20].

Obwohl der PREHO-LYO-Trial keinen Unterschied in der Therapie von Traumapatienten bei Anwendung von lyophilisiertem Plasma vs. 0,9 %iger NaCl-Lösung nachweisen konnte – weder in Bezug auf den INR-Wert noch auf die 30-Tage-Mortalität – konnte gezeigt werden, dass die prähospitale Anwendung von lyophilisiertem Plasma möglich und sicher ist, da keine unerwünschten, transfusionsbedingten Nebenwirkungen auftraten [21]. Prähospital verabreichte Erythrozytenkonzentrate in Kombination mit lyophilisiertem Plasma konnten in der RePHILL-Studie keine Überlegenheit im zusammengesetzten primären Endpunkt (Krankenhaussterblichkeit oder Lactat-Clearance [> 20 % pro Stunde innerhalb der ersten 2 h]) im Vergleich zu 0,9 %iger NaCl-Lösung nachweisen [22]. Die Interventionsgruppe hatte bei Krankenhausaufnahme eine signifikant höhere Hämoglobinkonzentration und erhielt im Laufe der Behandlung mehr Blutprodukte.

Der Umbrella-Review von Gianola et al. zeigte eine geringe 30-Tage-Mortalität (1 RCT: RR = 0,51) bei gleichzeitiger Anwendung von Plasma und Erythrozytenkonzentraten im Vergleich zur Standardtherapie. Präklinisch verabreichtes Plasma reduzierte die 24-h-Sterblichkeit bei Patienten im hämorrhagischen Schock (2 RCT: RR = 0,69) [23].

Zusammenfassend belegen die bisherigen Studien, dass die prähospitale Gabe von Plasma grundsätzlich möglich und sicher ist. Es besteht jedoch weiterer Forschungsbedarf, um die Patientengruppen, welche tatsächlich davon profitieren, zu identifizieren. Nach aktuellem Stand gibt es insbesondere in Regionen mit kurzen Transportzeiten bislang keinen überzeugenden Hinweis auf einen Vorteil der präklinischen Plasmatransfusion.

#### Innerklinische Behandlung

Stumpfe und penetrierende Traumata sind die häufigsten Ursachen für hämorrhagische Schockzustände, gefolgt von chirurgischen bzw. perioperativen Blutungen sowie Blutungen aus dem Magen-Darm-Trakt. Rezente Daten deuten darauf hin, dass die frühe Transfusion von Blutprodukten bei Patienten im hämorrhagischen Schock der Praxis einer kristalloidbasierten Infusionstherapie überlegen ist [24]. Das ideale Verhältnis von Blutprodukten im Rahmen der Massivtransfusion ist noch Gegenstand aktueller Forschung. Ein Mortalitätsvorteil wurde bei einem Transfusionsverhältnis von Erythrozyten:Plasma:Thrombozyten zwischen 1:1:1 und 2:1:1 (bei Einzelspenderthrombozytenkonzentraten: 4:4:1 bzw. 8:4:1) im Vergleich zu einer Transfusion mit einem niedrigeren Plasma-zu-Erythrozyten-Verhältnis nachgewiesen [23, 25]. Dies wird ebenfalls in der aktuellen Empfehlung der „*European Guideline on Management of Major Bleeding and Coagulopathy Following Trauma*“ deutlich: Für die initiale Stabilisierung bei akut blutenden Patienten wird ein Verhältnis von FFP zu Erythrozyten von mindestens 1:2 empfohlen [24].

Beim blutenden Traumapatienten soll die Gerinnung so früh wie möglich kontrolliert werden – idealerweise bereits direkt bei Aufnahme im Schockraum. Dies kann mittels viskoelastischem Monitoring (Rotationsthrombelastometrie [ROTEM], Thrombelastographie [TEG]) oder durch Laboranalysen erfolgen, um eine traumainduzierte Koagulopathie frühzeitig zu erkennen und gezielt behandeln zu können [24, 26]. Die Autoren empfehlen den Einsatz von Point-of-Care-Testing wie ROTEM/TEG aufgrund der raschen Verfügbarkeit von Ergebnissen, der umfassenden Gerinnungsbeurteilung (einschließlich Clotting Time, Maximum Clot Firmness und Fibrinolyseaktivität) sowie der Möglichkeit einer zielgerichteten Therapie, wodurch nicht nur eine effektive Behandlung, sondern auch die Vermeidung einer Übertherapie ermöglicht wird. Moderne Point-of-Care-Geräte ermöglichen bereits innerhalb von 5–15 min eine erste Beurteilung des Gerinnungsstatus.

Da ein nichtanämischer, blutender Patient nicht nur Erythrozyten, sondern auch 55–60 % seines verlorenen Blutvolumens in Form von Plasma (damit Gerinnungsfaktoren, Proteine und Flüssigkeit) verliert, ist es naheliegend, diesen Verlust durch Plasmagaben auszugleichen. Eine Studie an englischen Traumazentren zeigte jedoch, dass die durchschnittliche Zeit bis zur ersten Plasmagabe etwa 87 min (42,5–229 min; *n* = 300) beträgt (im Rahmen von Massivtransfusionen 68 min (30–131,5 min; *n* = 120)) [27]. Aufgrund von Auftauzeiten und logistischen Herausforderungen ist es oft schwierig, Plasma gleichzeitig mit dem ersten Erythrozytenkonzentrat zu verabreichen. Lyophilisiertes Plasma könnte hier die Dauer bis zur ersten Plasmagabe erheblich verkürzen.

Bis Ergebnisse von viskoelastischen bzw. laborchemischen Tests vorliegen, kann alternativ eine Gabe von 2 g Fibrinogen [24] bzw. 30–60 mg/kgKG [26] gemeinsam mit den ersten 4 Erythrozytenkonzentraten erfolgen. Dies soll ein 1:1-Verhältnis von Plasma zu Erythrozyten simulieren und frühzeitig einer Hypofibrinogenämie vorzubeugen – da Fibrinogen als „first factor down“ gilt. Da Plasma Fibrinogen nur in physiologischer Konzentration enthält, ist es wenig effektiv, einen ausgeprägten Fibrinogenmangel rasch zu beheben [24]. Bei innerklinischer Behandlung traumainduzierter Koagulopathien erwiesen sich Gerinnungsfaktorkonzentrate als effektivere First-line-Therapie im Vergleich zu FFP. Patienten, die mit FFP behandelt wurden, benötigten häufiger Massivtransfusionen (30 % vs. 12 %) und Rescue-Therapien (52 % vs. 4 %; Rescue-Therapie bei FFP-Behandlung: nach 2‑maliger Applikation von 15 ml Plasma pro kgKG erhielten Patienten die Vergleichstherapie mit Gerinnungsfaktorkonzentraten). Die frühzeitige Verabreichung von Fibrinogen bei Patienten mit Polytrauma erwies sich bei schweren Gerinnungsstörungen als vorteilhaft [28].

Die initiale Volumentherapie bei blutenden, traumatisch verletzten Patienten soll (sparsam) durch balancierte kristalloide Infusionen mit dem Ziel einer permissiven Hypotonie (Ausnahme: Schädel-Hirn-Trauma) erfolgen. Bleibt der Patient weiterhin instabil, können Kolloide (Humanalbumin, Gelatine) verwendet werden [29].

Die Verabreichung großer Mengen kristalloider oder kolloidaler Infusionen kann das Auftreten einer Verdünnungskoagulopathie fördern. Kolloide können zusätzlich die Fibrinpolymerisation und die Thrombozytenaggregation negativ beeinflussen [30]. Bei hämodynamisch instabilen Patienten (Blutungen der Klassen III und IV laut ATLS-Score) sowie unkontrollierten, anhaltenden Blutungen sollen Blutprodukte eingesetzt werden sollen [29].

### Substitution von Gerinnungsfaktoren

Plasma kann prinzipiell zur Substitution von Gerinnungsfaktoren eingesetzt werden, wenn die Aktivität mehrerer Gerinnungsfaktoren gleichzeitig erniedrigt ist und kein Gerinnungsfaktorenkonzentrat zur Verfügung steht. Bei der Substitution von Gerinnungsfaktoren mittels Plasmaprodukten gilt es stets zu bedenken, dass die Konzentration der Gerinnungsfaktoren in den einzelnen Plasmaprodukten zumeist nur subphysiologisch ist und daher große Transfusionsvolumina erforderlich sind, um die Faktorenkonzentration bzw. -aktivität beim Patienten zu steigern. Daher sind – egal, ob beim blutenden oder nichtblutenden Patienten – Gerinnungsfaktorenkonzentrate effektiver als Plasma, um die Aktivität bzw. Konzentration der Gerinnungsfaktoren anzuheben. Grundsätzlich stehen Einzelfaktorenkonzentrate mit Ausnahme von Faktor V und Faktor XI für alle Gerinnungsfaktoren kommerziell zur Verfügung. Zum Ausgleich von Vitamin-K-abhängigen Gerinnungsfaktoren stehen 4‑Faktoren-Konzentrate (z. B. PPSB-Komplex) zur Verfügung. Letztere waren Plasmaprodukten bei dieser Indikation im Risiko-Nutzen-Profil (90-Tages-Mortalität [OR 0,60; *p* = 0,01], bessere INR-Reversierung [OR 7,36; *p* < 0,00001] und weniger therapieassoziierte Nebenwirkungen [OR 0,45; *p* = 0,006]) deutlich überlegen [31].

Werden Plasmaprodukte zur Substitution von Gerinnungsfaktoren verwendet, ist angesichts der oben erwähnten niedrigen Faktorenkonzentration im Plasma eine adäquate Dosierung erforderlich. Diese beträgt mindestens 30 ml/kgKG [4] und erklärt, warum gerade bei nichtblutenden Patienten die Plasmatransfusion in dieser Indikation mit einer hohen Komplikationsrate (z. B. TACO) assoziiert ist.

## Obsolete Plasmaanwendungen

Neben den genannten evidenzbasierten Anwendungen wird Plasma auch in anderen Bereichen eingesetzt, obwohl diese Nutzung nicht durch Daten gestützt ist oder sogar widerlegt wurde. Die Auswertung verabreichter Plasmaprodukte im Jahr 2017 in Ontario (Kanada) zeigte, dass in 77,7 % der Fälle eine Plasmatransfusion aufgrund einer fraglichen Indikation durchgeführt wurde. In 34 % der Fälle lag keine geeignete Indikation vor, und in 70,7 % der Fälle wurden subtherapeutische Dosen von Plasma verabreicht. Davon wurde in 25,2 % der Fälle lediglich eine einzelne Einheit Plasma verabreicht [32].

### Prophylaktische Plasmatransfusion

In der klinischen Praxis werden Plasmaprodukte immer wieder aufgrund von verlängerten laborchemischen Gerinnungszeiten (z. B. Prothrombin- oder partielle Thromboplastinzeit) prophylaktisch vor invasiven Maßnahmen (beispielsweise Tracheotomien, ZVK-Anlage, Operationen) verabreicht. Grundsätzlich ist festzuhalten, dass kein Zusammenhang zwischen erhöhtem Blutungsrisiko und einer verlängerten Prothrombin- oder partiellen Thromboplastinzeit besteht. Beide Gerinnungsanalysen wurden entwickelt, um den Gerinnungseffekt von Vitamin-K-abhängigen Antikoagulanzien bzw. unfraktioniertem Heparin laborchemisch monitorisieren zu können. Entsprechend sind diese Parameter auch ungeeignet, um das Blutungsrisiko vor oder nach einem invasiven Eingriff abschätzen zu können. Deshalb können sie auch nicht herangezogen werden, um die prophylaktische Gabe von Gerinnungsprodukten vor oder nach solchen Eingriffen zu indizieren.

Studiendaten zeigen, dass die periprozedurale Transfusion von Plasma bei kritisch kranken Patienten nicht notwendig ist. Durila et al. berichteten, dass eine Tracheotomie bei unauffälliger „coagulation time“ in der Thrombelastometrie trotz erhöhter INR-Werte ohne erhöhtes Blutungsrisiko durchgeführt werden kann [33].

### Leberinsuffizienz

Leberfunktionsstörungen oder -versagen bei kritisch kranken Patienten sind oft mit einer laborchemischen Gerinnungsstörung assoziiert. Die Leber spielt eine zentrale Rolle bei der Bildung sowohl pro- als auch antikoagulatorischer Faktoren. Konventionelle Gerinnungstests können daher das Blutungsrisiko bei Patienten mit chronischen Lebererkrankungen nicht vorhersagen und sollten nicht zur Therapieentscheidung herangezogen werden [34].

Bei Eingriffen mit niedrigem Blutungsrisiko muss eine laborchemisch auffällige Gerinnung nicht korrigiert werden. Im Rahmen von aktiven Blutungen oder Eingriffen mit besonders hohem Blutungsrisiko können viskoelastische Testmethoden unterstützen.

Um die Aktivität bzw. Konzentration von Gerinnungsfaktoren mit Plasmaprodukten ausreichend zu steigern, sind große Mengen erforderlich (s. oben). Diese Volumina wiederum erhöhen das Risiko für das Auftreten eines TACO und steigern außerdem den Druck in den Portalgefäßen, was wiederum zu (Nach)Blutungen im oberen Gastrointestinaltrakt führen kann [35].

## Kontraindikationen

Eine Kontraindikation zur Anwendung von Plasma liegt bei bekannter Plasmaunverträglichkeit sowie nachgewiesenem IgA-Mangel vor. Der angeborene IgA-Mangel liegt mit einer Prävalenz von 1:650 vor und kann mit dem Vorhandensein von Anti-IgA-Antikörpern einhergehen. Diese Antikörper wurden mit anaphylaktischen Reaktionen nach der Gabe von IgA-haltigen Blutprodukten in Verbindung gebracht. Allerdings ist dieser Zusammenhang umstritten [4].

## Zukünftige Anwendungsgebiete

Plasma könnte zukünftig auch abseits der obigen genannten Indikationen eingesetzt werden, allerdings sind hierfür noch hochwertige Studien zur Validierung erforderlich. Derzeit gibt es in den aktuellen Leitlinien keine Empfehlung zur Plasmatherapie bei Sepsis oder Verbrennungen. Bisher ist auch unklar, welche Patientengruppen tatsächlich davon profitieren könnten. Plasmaprodukte vermitteln nicht nur einen nachhaltigen intravasalen Volumeneffekt, der gerade bei Patienten im hämorrhagischen Schock Vorteile bringt, sondern schützen auch die endotheliale Glykokalyx und reduzieren somit die transkapilläre Permeabilität [36]. Dieser pleiotrope Effekte könnte auch für Patienten außerhalb der Traumatologie vorteilhaft sein.

### Sepsis und septischer Schock

Patienten im septischen Schock werden oft mit kristalloiden Infusionslösungen behandelt; diese können jedoch einen Endothelschaden durch negative Auswirkungen auf die endotheliale Glykokalyx auslösen oder verschlimmern. Dadurch kommt es vermehrt zu Flüssigkeitsüberladung, was Ödeme fördert sowie die Organdysfunktion und die Mortalität erhöht. Plasma könnte durch seine protektiven Eigenschaften auf die endotheliale Glykokalyx nicht nur den Flüssigkeitsbedarf von Sepsispatienten, sondern auch die Ödembildung und Organdysfunktion reduzieren [36]. Trotz dieser Erkenntnisse zeigte sich bei Behandlung einer sepsisinduzierten Koagulopathie mit Plasma eine erhöhte Krankenhaussterblichkeit (OR 1,44; 95 %-KI 1,23–1,69; *p* < 0,05) [37]. Die Surviving Sepsis Campaign spricht sich in ihrer Guideline weder für noch gegen die Anwendung von Plasma aus [38]. Ein therapeutischer Plasmaaustausch bei septischen Patienten könnte im Vergleich zur Standardtherapie die Kurzzeitsterblichkeit senken (RR 0,59; 95 %-KI 0,47–0,74) [39]. Allerdings dürfte dieser Therapieansatz nur für eine begrenzte Patientengruppe von Bedeutung sein.

### Verbrennungsschock

Von ähnlichen Effekten könnten auch Patienten mit großflächigen Verbrennungen (> 20 % Körperoberfläche) profitieren [40]. Die Flüssigkeitstherapie ist essenzieller Bestandteil in der Behandlung dieses Krankheitsbildes. Plasma könnte dazu beitragen, Volumen effektiv zu ersetzen und gleichzeitig endothelialen Schäden entgegenzuwirken [41]. In einer retrospektiven Datenanalyse zeigte sich bei Patienten mit Verbrennungen von mehr als 30 % der Körperoberfläche eine signifikant niedrigere Mortalität unter Behandlung mit FFP im Vergleich zu Albumin (33,8 % vs. 78,9 %; *p* < 0,0007) [42]. Obwohl die Studie erst 2023 veröffentlicht wurde, stammen die zugrunde liegenden Daten aus den Jahren 2006–2012, was die Aussagekraft im aktuellen Kontext einschränken könnte.

In der aktuellen Ausgabe der Leitlinie zur *Behandlung thermischer Verletzungen bei Erwachsenen (2021)* wird allerdings, basierend auf den Empfehlungen der Bundesärztekammer Deutschland, von der Verwendung von FFP zur Volumensubstitution abgeraten [4, 43].

## Fazit für die Praxis

- Plasma soll grundsätzlich AB0-gleich transfundiert werden – im Notfall ist auch eine AB0-kompatible Transfusion möglich.
- Plasma der Blutgruppe AB gilt als Universalspenderplasma, umgekehrt ist die Blutgruppe 0 Universalempfänger.
- Die Transfusion erfolgt über ein Infusionsbesteck mit Standardfilter (Porengröße 170–230 μm).
- Die aktuelle Evidenz unterstützt die Plasmaanwendung bei Massivblutungen zum Plasmaersatz sowie zur Substitution einzelner oder mehrerer Gerinnungsfaktoren, wenn keine Einzelfaktorkonzentrate verfügbar sind. Dies betrifft vor allem Faktor V und Faktor XI da hier keine Einzelfaktorkonzentrate verfügbar sind.
- Eine prophylaktische Plasmatransfusion vor invasiven Eingriffen oder zur Korrektur von Gerinnungslaborparametern bei Leberinsuffizienz soll nicht erfolgen.
- Zu den häufigsten Nebenwirkungen zählen die iatrogene Hypokalzämie (Plasma enthält viel Citrat) sowie das Risiko einer transfusionsbedingten Hypervolämie (TACO „Transfusion-associated circulatory overload“).
